# Predicting reduced left atrial appendage velocity from echocardiographic left atrial function parameters in patients with atrial fibrillation undergoing catheter ablation

**DOI:** 10.1038/s41598-024-57947-6

**Published:** 2024-03-27

**Authors:** Beata Uziębło-Życzkowska, Marek Kiliszek, Małgorzata Maciorowska, Magdalena Potapowicz-Krysztofiak, Krystian Krzyżanowski, Agnieszka Jurek, Paweł Krzesiński

**Affiliations:** Department of Cardiology and Internal Diseases, Military Institute of Medicine–National Research Institute, Warsaw, Poland

**Keywords:** Cardiovascular diseases, Atrial fibrillation

## Abstract

Decreased left atrial appendage velocity (LAAV) is considered a significant risk factor thrombus formation in the left atrial appendage (LAA). The aim of this study was to assess the role of echocardiographic left atrial (LA) function parameters in predicting LAAV in patients with persistent atrial fibrillation (AF) undergoing catheter ablation. We prospectively enrolled consecutive patients with persistent AF undergoing transesophageal echocardiography (TEE) directly before the first AF ablation in 2019–2022. Of the 150 patients enrolled in the study, 29.3% (n = 44) had reduced LAAV values defined as < 25 cm/s. Patients with decreased LAAV values exhibited significantly reduced left atrial reservoir and conduit strain (LASr and LAScd), LA emptying fraction, and average e′ values. This group also presented with a high LA stiffness index (LASI), high LA and right atrial area, and high LA volume index (LAVI) and E/e′ ratio. In multivariable logistic regression analysis, LASI and LAVI remained significant predictors of the reduced LAAV. The threshold values were 1.6 for LASI and 44.47 ml/m^2^ for LAVI, with area under the curve values of 0.809 and 0.755, respectively. Among all noninvasive echocardiographic parameters, LASI and LAVI were found to be the best predictors of reduced LAAV, with good sensitivity and specificity. Moreover, LASI was found to be the only significant predictor of reduced LAAV defined as < 20 cm/s as well as < 25 cm/s.

## Introduction

Left atrial appendage (LAA) is considered the most common site of thrombus formation. Left atrial appendage emptying velocity (LAAV) assessed by Doppler tracking of LAA flow is the most commonly used parameter to assess the mechanical function of LAA. Numerous studies have confirmed that decreased LAAV is considered a significant risk factor for thrombus formation, especially in the LAA^1–3^. Reduced LAAV in transesophageal echocardiography (TEE) has also been shown to be associated with multiple infarcts in patients with cryptogenic stroke^4^. The LAAV is not only a simple marker of blood flow but also a reflection of the mechanical and endocrine functions of LAA. The LAA is involved in the regulation of left atrial pressure, as its distensibility is greater than that of the left atrium (LA)^5^. In addition, atrial natriuretic peptide concentrations are highest in the LAA, which by affecting heart rate, diuresis, and natriuresis, further helps modulate LA pressure^5^. All these factors could modulate LAAV. Therefore the LAA is also regarded as one of the important sources of atrial fibrillation (AF)^6^. Ueno et al.^7^ revealed that LAAV < 37.5 cm/s could predict AF detected with an insertable cardiac monitor in patients with cryptogenic stroke. Two large meta-analyses have also shown that LAAV is one of the factors that increases the likelihood of AF recurrence after catheter ablation^8,9^. Raval et al.^10^ reported that LAAV ≤ 30 cm/s was independently associated with an increased risk of AF recurrence after TEE-guided electrical cardioversion. In our recent study, LAAV below 44 cm/s was found to be an independent predictor of AF recurrence after catheter ablation^11^.

LAAV can easily be calculated using Doppler during TEE, which is the first technique of choice for assessing LAAV in clinical practice. However, TEE is a semi-invasive examination and is very unpleasant for patients. Moreover, it can cause complications. Therefore, it seems justified to search for predictors of reduced LAAV using TTE, which is noninvasive and more common in everyday clinical practice. Since every AF patient has an indication for TTE, only a few have an indication for TEE.

Consequently, our aim was to assess the role of echocardiographic LA function parameters in predicting LAAV in patients with persistent atrial fibrillation undergoing catheter ablation.

## Materials and methods

### Study design and patients

The study group consisted of consecutive patients with persistent AF admitted to the cardiology department from 2019 to 2022 for a first-time ablation of AF. This was a prospective, observational, and single-center study. Patients with sinus rhythm on echocardiography had high LAAV values (median [IQR]: 54.5 cm/s [38–74]) and were not included in the analysis. All patients underwent conventional and 2D speckle-tracking echocardiography on the day of ablation. The heart rhythm (AF vs. sinus rhythm) was the same during echocardiography and ablation procedure in all included patients. Exclusion criteria for the study were presence of valvular AF (defined as moderate or severe mitral stenosis and artificial mitral valve), age over 80 years, status after percutaneous left atrial appendage closure, and poor echocardiographic image quality. No other exclusion criteria were used.

### Transthoracic and transesophageal echocardiography

In this study, TTEs were performed on the day of ablation using high-quality echocardiographs (Vivid E95, General Electric, United States). All examinations were analyzed offline (using GE EchoPAC BT12) by a single experienced echocardiographer (blinded to clinical status) with expert status in TTE and TEE conferred by the Echocardiography Section of the Polish Cardiac Society. All conventional echocardiographic parameters, as well as LA and LV deformation measurements were obtained, as recommended in the current guidelines of the European and American Societies of Cardiology^12,13^. The QRS wave onset was set as a reference point for assessing the LA reservoir strain value (LASr). Left atrial strain analysis was performed using automated software specifically dedicated to LA assessment (automated functional imaging of the left atrium [AFI LA]). The LA emptying fraction (LAEF) was determined automatically using AFI LA. For all strain measurements, while some segments were excluded because of the inability to achieve adequate tracking, global strain values were calculated from the average values measured in the remaining segments. The left atrial stiffness index (LASI) was assessed noninvasively and was defined as the E/e′ ratio/LASr. Measurement of the LAA emptying velocity was taken 1 cm from the LAA ostium and averaged from three measurements at two different scanning angles.

All patients were classified into two groups according to different values of LAAV: the reduced LAAV group (< 25 cm/s) and the high LAAV group (≥ 25 cm/s). The LAAV value of < 25 cm/s was chosen based on the results of previous studies, which have shown it to be a useful cut-off value for discriminating patients at high risk of systemic embolism^14^, as well as being the threshold used in other relevant studies^15^. A value of ≤ 20 cm/s was not adopted because of the small number of patients with these values in our study. However, a subgroup analysis was performed for patients with LAAV below and above 20 cm/s due to the recognised value of LAAV < 20 as significantly increasing the risk of LAA thrombus.

### Statistical analysis

The distribution and normality of continuous variables were assessed using the Shapiro–Wilk test. Continuous variables were presented as means (standard deviation [SD]) or medians (interquartile range [IQR]: 1st–3rd quartile) depending on their distribution. Categorical variables were presented as absolute and relative values (percentages). Independent t-tests, Mann–Whitney U tests, and chi-square tests were applied to compare two groups of continuous and categorical variables. A two-tailed p-value of < 0.05 was considered statistically significant. To identify the predictors of reduced LAAV, univariable and multivariable logistic regression analyses were performed. While selecting variables for the univariable and multivariable models, we were guided by variables that were statistically significant, that is, those that differentiated the groups of patients being compared (reduced and high LAAV values). If highly correlated parameters were present, only one representative was chosen for the multivariable analysis based on its p-value in the univariable analysis and its biological validity. Receiver operating characteristic (ROC) curves with area under curve (AUC) were calculated to estimate the cut-off value for the parameters revealed to be significant by multivariable logistic regression analysis. Statistica 13.0 statistical software (StatSoft Inc., Tulsa, OK, USA) was used for all the analyses.

### Ethical approval

The studies were conducted in compliance with the Declaration of Helsinki. The study protocol was approved by the Ethics Committee of the Military Institute of Medicine in Warsaw (50/WIM/2019) and informed consent was obtained from all patients.

## Results

A total of 167 patients enrolled for this study. Seventeen patients were excluded from the analysis including 7 with poor visualization on echocardiography, 7 with post-percutaneous left atrial appendage closure, and 3 with valvular AF (1 with moderate mitral stenosis and 2 with artificial mitral valve). The final analysis included 150 patients with persistent AF undergoing catheter ablation. The median age for the entire group was 66.5 years (IQR 57–71), the majority of the study population was male (101 patients (67.3%)). A large part of the study group was either overweight (37%) or obese (52%). The most common comorbidities in the study population were hypertension (76.7%) and heart failure (38%). The median value of LAAV in the entire group was 31.5 cm/s (IQR 24–42). Of all study patients, 44 had LAAV < 25 cm/s and 106 patients had LAAV ≥ 25 cm/s. These subgroups did not differ in terms of age, gender, body mass index (Table 1), or comorbidities (Table 2).

**Table 1:** Baseline characteristics of the two groups of study patients.

Variables	AF group		
	LAAV < 25 cm/s n = 44	LAAV ≥ 25 cm/s n = 106	p
LAAV (cm/s), median (IQR)	21.5 (19–23)	36.5 (30–48)	
Demographic data			
Time from first AF diagnosis years, median (IQR)	4 (2–7)	3 (1–5)	0.04
Age, years, median (IQR)	67.5 (61.5–74)	65 (56–71)	0.09
Female gender, n (%)	18 (40.9)	31 (29.2)	0.26
BMI, median (IQR)	30.1 (26.5–31.5)	30.8 (27.1–34.2)	0.14
Echocardiographic data, median (IQR)			
LVDd, mm	51 (47–56.5)	49 (45–54)	0.06
LVMI, g/m^2^	135.8 (110–162)	125 (103.2–144.2)	0.02
e′ average, cm/s	7 (5.5–9)	9 (8–10.5)	< 0.001
E/e′ average	12.8 (10.1–16)	8.7 (7.3–10.7)	< 0.001
LVEF, %	51 (36–59)	56 (50–61)	0.009
LA area, cm^2^	31.4 (28.1–35)	27.8 (24.4–32.2)	0.002
RA area, cm^2^	22.8 (19.5–26)	20.1 (17.3–22.7)	0.004
LAVI, ml/m^2^	56.1 (46.8–72.6)	43.2 (36.8–53.3)	< 0.001
LAEF, %	23 (15–27)	30 (24–36)	< 0.001
Speckle tracking echocardiography data—left atrial and left ventricular function parameters			
LASr, %, mean (SD)	7.4 (2.97)	10.7 (3.33)	< 0.001
LV GLS, %, median (IQR)	12 (8.4–15.4)	14 (12–16.1)	0.004
LASI (E/e′/LASr), median (IQR)	1.9 (1.1–2.6)	0.85 (0.6–1.2)	< 0.001

AF, atrial fibrillation; BMI, body mass index; LA, left atrium; LAAV, left atrial appendage emptying velocity; LAEF, left atrial emptying fraction; LASI, left atrial stiffness index (E/e′/LASr); LASr, left atrial strain during reservoir phase; LAVI, left atrial volume index; LVDd, left ventricular diastolic diameter; LVEF, left ventricular ejection fraction; LV GLS, left ventricular global longitudinal strain; LVMI, left ventricular mass index; RA, right atrial.

**Table 2:** Clinical data of the two study groups of patients.

Variables	AF group		
	LAAV < 25 cm/s n = 44	LAAV ≥ 25 cm/s n = 106	p
Clinical data, n (%)			
Heart failure	21 (47.7)	37 (34.9)	0.187
HFrEF	12 (27.3)	14 (13.2)	0.071
HFmrEF	5 (11.4)	12 (11.3)	0.998
HFpEF	4 (9.1)	11 (10.4)	0.566
Hypertension	31 (70.5)	84 (79.2)	0.399
Diabetes mellitus	15 (34.1)	15 (14.2)	0.055
Transient ischemic attack/stroke	2 (4.5)	7 (6.6)	0.829
Myocardial infarction	3 (6.8)	10 (9.4)	0.804
Coronary artery disease	10 (22.7)	28 (26.4)	0.725
Vascular disease	11 (25)	28 (26.4)	0.894
Chronic kidney disease	13 (29.5)	18 (17)	0.228
Smoking	8 (18.2)	23 (21.7)	0.737
Hypothyroidism	6 (13.6)	10 (9.4)	0.688
CHA_2_DS_2_VASC, median (IQR)	3 (2–4)	2 (1–4)	0.051
Treatment data, n (%)			
VKA	3 (6.8)	2 (1.9)	0.151
NOAC	41 (93.2)	104 (98.1)	0.151
Amiodarone	5 (11.4)	13 (12.3)	0.877
Verapamil	0	0	1
ASA	2	1	0.206
Other antiplatelet drugs	3	3	0.359
Steroids	0	2	0.892
Nonsteroids drugs	0	0	1

HFmrEF, heart failure with mildly reduced ejection fraction; HFpEF, heart failure with preserved ejection fraction; HFrEF, heart failure with reduced ejection fraction; LAAV, left atrial appendage emptying velocity.

Both groups showed significant differences in LA pressures measured directly during ablation and the presence of low-voltage areas in the LA, as detailed in Table S1. These results were not analyzed in the study, as the purpose of the study was to estimate reduced LAAV values based on a non-invasive evaluation performed before the ablation procedure.

The reduced LAAV (< 25 cm/s) subgroup had longer duration of arrhythmia, significantly higher LA volume index (LAVI), LASI, and E/e′ and significantly lower LASr, LAEF, and e′ compared to the higher LAAV (≥ 25 cm/s) subgroup. Details of the differences in the parameters assessed between the two study groups are shown in Tables 1 and 2.

In the univariable logistic regression analysis, 14 variables (including 11 echocardiographic parameters) were associated with reduced LAAV values (Table 3). However, in the multivariable logistic regression analysis, only LASI and LAVI remained independent predictors (Table 3).

**Table 3:** Univariable and multivariable logistic regression analysis.

Variables	Univariable analysis			Multivariable analysis		
	p-value	Odds ratio (OR)	OR 95% CI	p-value	Odds ratio (OR)	OR 95% CI
Time from first AF diagnosis, years	0.04	1.09	1.002–1.186			
Age, years	0.06	1.039	0.998–1.081			
Gender	0.17	1.675	0.805–3.484			
LVMI, g/m^2^	0.94	1.000	0.998–1.002			
LASr, %	< 0.001	0.711	0.617–0.819			
LASI	< 0.001	3.804	2.041–7.090	0.005	3.065	1.415–6.639
LA area, cm^2^	0.003	1.105	1.036–1.179			
RA area, cm^2^	0.003	1.123	1.042–1.211			
LAVI, ml/m^2^	< 0.001	1.067	1.038–1.097	0.01	1.050	1.011–1.091
LAEF, %	< 0.001	0.888	0.844–0.935			
LV GLS, %	0.002	0.849	0.765–0.942			
e′ average, cm/s	< 0.001	0.643	0.528–0.784			
E/e′ average	< 0.001	1.307	1.169–1.461			
LV EF, %	0.001	0.948	0.918–0.979			

AF, atrial fibrillation; LA, left atrial; LAEF, left atrial emptying fraction; LASI, left atrial stiffness index (E/e′/LASr); LASr, left atrial strain during reservoir phase; LAVI, left atrial volume index; LVEF, left ventricular ejection fraction; LV GLS, left ventricular global longitudinal strain; LVMI, left ventricular mass index; RA, right atrial.

**Figure 1:**
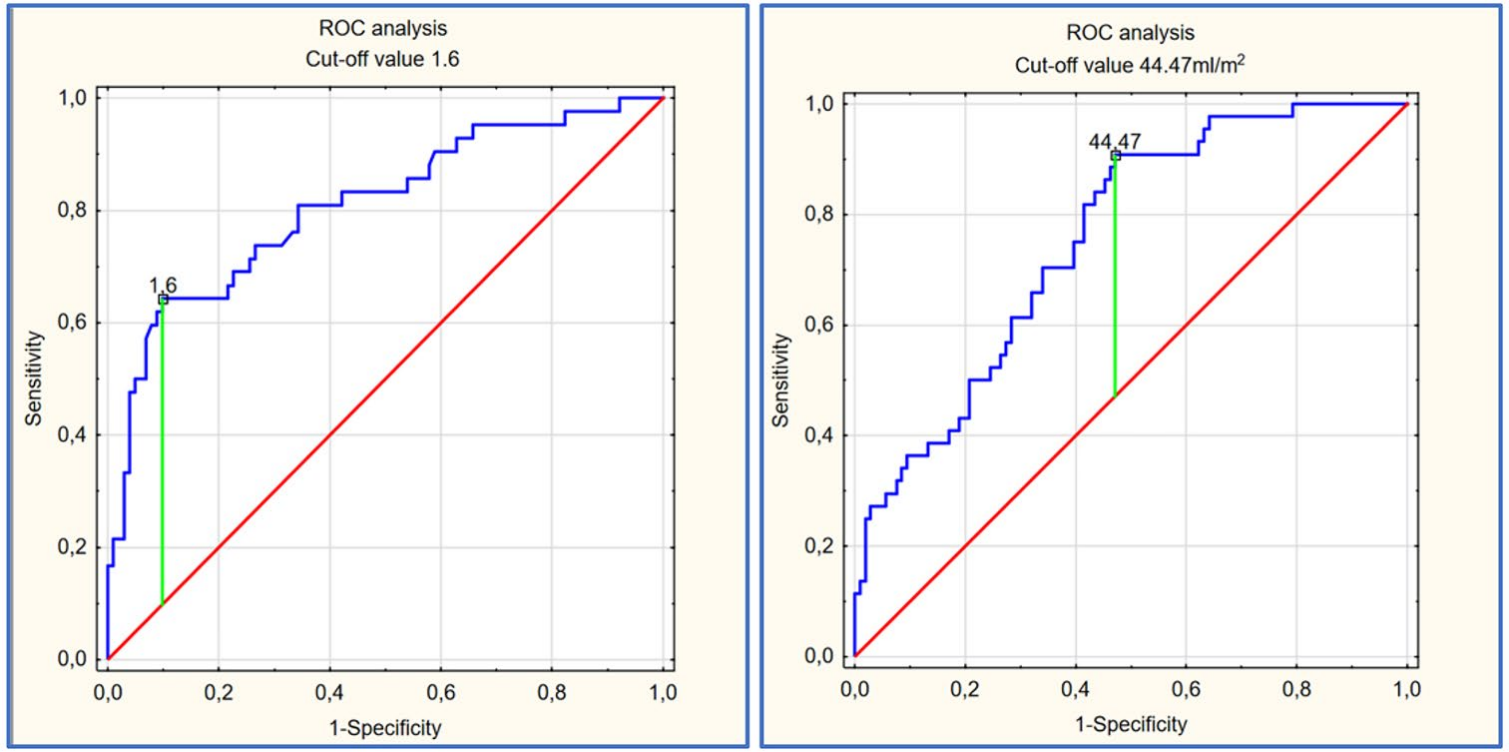
ROC analysis in predicting reduced LAAV for LASI (left) and LAVI (right).

The diagnostic accuracy of these two parameters in predicting reduced LAAV was high. The AUC value for LASI and LAVI was 0.809 (95% CI 0.726–0.892) and 0.755 (95% CI 0.676–0.835), respectively. An LASI threshold of 1.6 presented with 64% sensitivity and 90% specificity and an LAVI threshold of 44.47 ml/m^2^ presented with 91% sensitivity and 53% specificity in predicting reduced LAAV defined as < 25 cm/s (Fig. 1).

A score was created from both of the parameters (LAVI and LASI), awarding the presence of each a point. The sensitivity and specificity for predicting reduced LAAV, when both parameters were present (LASI > 1.6 and LAVI > 44.47 ml/m^2^), were 55% and 93%, respectively (see Table 4 for details).

**Table 4.** Sensitivity and specificity of LASI and LAVI considered together and separately in predicting reduced LAAV (< 25 cm/s).

Variables	Sensitivity (%)	Specificity (%)
LASI > 1.6	64	90
LAVI > 44.47 ml/m^2^	91	53
0 pkt: neither LAVI > 44.47 ml/m^2^ nor LASI > 1.6	7	50
1 point: LAVI > 44.47 ml/m^2^ or LASI > 1.6	39	57
2 points: LAVI > 44.47 ml/m^2^ and LASI > 1.6	55	93

LAAV, left atrial appendage velocity; LASI, left atrial stiffness index (E/e′/LASr); LAVI, left atrial volume index.

A subgroup analysis comparing patients with LAAV below and above 20 cm/s revealed that patients with LAAV < 20 cm/s presented significantly higher LA area, LAVI, LASI, and E/e′ and significantly lower LASr and LAEF compared to those with higher LAAV ≥ 20 cm/s. In this analysis, statistical significance was not reached by the single parameter e' in relation to the division of groups according to LAAV 25 cm/s. The detailed results of this analysis are presented in Tables 5 and 6.

**Table 5:** Results of comparative analysis of groups divided according to the LAAV cut-off of 20 cm/s—demographic and echocardiographic data.

Variables	AF group		
	LAAV < 20 cm/s n = 14	LAAV ≥ 20 cm/s n = 136	p
LAAV (cm/s), median (IQR)	18 (17–19)	34 (27–43)	
Demographic data			
Time from first AF diagnosis years, median (IQR)	4.5 (2.75–11)	3 (1–5)	0.061
Age, years, median (IQR)	67 (60–76)	66 (57–71)	0.554
Female gender, n (%)			0.773
BMI, median (IQR)	28.2 (27.1–30.9)	30.5 (26.9–33.9)	0.131
Echocardiographic data, median (IQR)			
Heart rhythm	101 (90–110)	105 (87–120)	0.777
LVDd, mm	50.5 (47–58)	50 (46–54)	0.584
LVMI, g/m^2^	132.2 (120.9–180.3)	127.3 (104.1–150.9)	0.132
e′ average, cm/s	6 (5–10)	8.5 (7–10)	0.075
E/e′ average	14.4 (8.3–19.4)	9.3 (7.5–11.7)	0.013
LAEF, %	18 (14–24)	28 (22–35)	0.002
LA area, cm^2^	32.3 (29.6–34.1)	28.3 (24.8–33.6)	0.026
RA area, cm^2^	23.4 (18.8–28.5)	21 (17.6–24)	0.075
LAVI, ml/m^2^	58.3 (49.9–67.1)	46.4 (37.4–59)	0.006
LVEF, %	54.5 (33–62)	55 (45–60)	0.680
Speckle tracking echocardiography data—left atrial and left ventricular function parameters			
LASr, %, mean (SD)	6.71 (3.099)	10 (3.47)	0.002
LV GLS, %, median (IQR)	12.4 (8.7–16.8)	13.7 (11.1–15.8)	0.520
LASI (E/e′/LASr), median (IQR)	2.23 (1.07–6.16)	0.96 (0.64–1.45)	< 0.001

AF, atrial fibrillation; BMI, body mass index; LA, left atrium; LAAV, left atrial appendage emptying velocity; LAEF, left atrial emptying fraction; LASI, left atrial stiffness index (E/e′/LASr); LASr, left atrial strain during reservoir phase; LAVI, left atrial volume index; LVDd, left ventricular diastolic diameter; LVEF, left ventricular ejection fraction; LV GLS, left ventricular global longitudinal strain; LVMI, left ventricular mass index; RA, right atrial.

**Table 6:** Results of comparative analysis of groups divided according to the LAAV cut-off of 20 cm/s—clinical and treatment data.

Variables	AF group		
	LAAV < 20 cm/s n = 14	LAAV ≥ 20 cm/s n = 136	p
Clinical data, n (%)			
Heart failure	6 (42.9)	51 (37.5)	0.775
HFrEF	4 (28.6)	17 (12.5)	0.111
HFmrEF	1 (7.1)	16 (11.8)	0.939
HFpEF	1 (7.1)	19 (13.9)	0.694
Hypertension	9 (64.3)	106 (77.9)	0.317
Diabetes mellitus	5 (35.7)	25 (18.4)	0.156
Transient ischemic attack/stroke	0 (0)	9 (6.6)	0.688
Myocardial infarction	1 (7.1)	12 (8.8)	0.831
Coronary artery disease	2 (14.3)	36 (26.5)	0.519
Vascular disease	2 (14.3)	37 (27.2)	0.522
Chronic kidney disease	4 (28.6)	27 (19.9)	0.489
Smoking	2 (14.3)	29 (21.3)	0.735
Hypothyroidism	0 (0)	16 (11.8)	0.365
CHA_2_DS_2_VASC, median (IQR)	2.5 (2–4)	3 (1.5–4)	0.781
Treatment data, n (%)			
VKA	3 (21.4)	4 (2.9)	0.069
NOAC	12 (85.7)	133 (97.8)	0.069
Amiodarone	3 (21.4)	15 (11)	0.378
Verapamil	0 (0)	0 (0)	1
ASA	0 (0)	3 (2.2)	0.744
Other antiplatelet drugs	1 (7.1)	5 (3.7)	0.450
Steroids	0 (0)	2 (1.5)	0.821
Nonsteroids drugs	0 (0)	0 (0)	1

HFmrEF, heart failure with mildly reduced ejection fraction; HFpEF, heart failure with preserved ejection fraction; HFrEF, heart failure with reduced ejection fraction; LAAV, left atrial appendage emptying velocity.

In this analysis, multivariable logistic regression results identified LASI as the only predictor of reduced LAAV defined as below 20 cm/s. ROC curve analysis indicated a cut-off point for LASI 1.73, which predicted reduced < 20 cm/s LAAV values with 67% sensitivity and 81% specificity (with AUC 0.778 (95% CI 0.633–0.923)). The results of this sub-analysis should be treated with caution due to the very small group with LAAV < 20 cm/s. Instead, they confirmed the significant importance of LASI as an echocardiographic predictor of reduced LAAV values.

Detailed results of univariable and multivariable logistic regression analysis for the LAAV below vs. above 20 cm/s groups are presented in Table 7.

**Table 7:** Univariable and multivariable logistic regression analysis for the LAAV below vs. above 20 cm/s groups.

Variables	Univariable analysis			Multivariable analysis		
	p-value	Odds ratio (OR)	OR 95% CI	p-value	Odds ratio (OR)	OR 95% CI
Age, years	0.517	1.020	0.960–1.085			
Gender	0.799	1.162	0.368–3.671			
LASr, %	0.002	0.727	0.596–0.887			
LASI	< 0.001	1.614	1.220–2.136	0.003	1.585	1.175–2.137
LA area, cm^2^	0.052	1.103	0.999–1.218			
LAVI, ml/m^2^	0.014	1.043	1.008–1.079			
LAEF, %	0.002	0.885	0.819–0.957			
E/e′ average	0.002	1.187	1.067–1.320			

## Discussion

The main purpose of this study was to evaluate the usefulness of noninvasive transthoracic echocardiography parameters in predicting reduced left atrial appendage emptying velocity. We showed that TTE-derived echocardiographic parameters (LASI and LAVI) appeared to be good predictors of reduced LAAV, defined as < 25 cm/s.

However, many other echocardiographic parameters remain interdependent predictors of impaired LAAV. This confirms the complex background of LAA hemodynamics and the interplay between LAA, LA, and LV. The relationship between LA morphology and hemodynamics has important clinical implications.

It is well known that LA enlargement is strongly associated with many cardiovascular diseases, including AF^16–18^, and this reduces the success of catheter ablation^19^. A large meta-analysis of 11 studies (1559 subjects) showed that patients with AF recurrences had a higher mean LA volume compared to those with no recurrences. Based on 9 other studies (1425 patients) comparing LAVI, it was shown that patients with AF recurrences had a higher mean LAVI compared to patients with no recurrence^19^. Increased LA dimensions were also associated with an increased risk of stroke in patients with AF^20^ and were an independent marker of recurrent cardioembolic or cryptogenic strokes in patients with first-ischemic stroke episodes^21^. Both reduced LAAV and increased LA size are considered risk factors for LAA thrombus and spontaneous echocontrast^22^.

We found that LA enlargement, assessed by LAVI, was a predictor of reduced LAAV values, and the cut-off point appeared to be 44.47 ml/m^2^. To date, the direct relationship between LA size and LAAV has been assessed in several studies. In a group of 716 patients with non-valvular AF, it was shown that enlarged LA was an independent risk factor for a decreased LAAV (defined as < 40 cm/s)^23^. Increased LA diameter was also one of the predictors of decreased LAAV in a study involving patients with nonvalvular paroxysmal AF and sinus rhythm during TTE^24^. However, in the aforementioned studies, LA size was assessed by the anteroposterior diameter of the LA, whereas the most accurate and established echocardiographic method for measuring LA size was the assessment of LA volume.

Our results are supported by Fukuhara et al.^25^, whose previous study showed a significant negative correlation between LAVI and LAAV; however, in their study, reduced LAAV was defined as < 35 cm/s and the study included patients with sinus rhythm.

Left atrial dilatation, best determined by LAVI, is the most common echocardiographic feature in patients with AF. As described by Leventopoulos et al.^26^, it is led by structural remodeling of the LA which is characterized by changes in atrial tissue properties and cellular ultrastructure. Atrial fibrosis plays a central role in the structural remodeling of the LA, and is mainly caused by the deposition of extracellular matrix proteins in the myocardial interstitial tissue. The transformation of fibroblasts into myofibroblasts leads to the development of atrial fibrosis and, consequently, not only structural but also electrical remodeling of the LA, as it causes prolongation of conduction and, as a result, contributing reentry and rotor formation in the LA^26^. In addition to MRI, echocardiographic LA strain assessment, is becoming an increasingly recognized non-invasive method for assessing the presence of areas of fibrosis in the LA. In particular, assessment of LA reservoir strain seems to particularly improve the diagnostic and prognostic value of previous conventional LA volume indices.

Left atrial stiffness index is a new non-invasive echocardiographic parameter that combines information from LASr and the E/e′ ratio. This parameter proved to be the strongest predictor of reduced LAAV values in our study and was the only one found to be a significant predictor of reduced LAAV defined as both < 25 cm/s and < 20 cm/s. This novel parameter can be assessed noninvasively using the intra-cavity filling pressure (E/e′ ratio) in conjunction with the percentage of maximal LA myocardial deformation (LASr)^27^. There was high agreement between LASI and the invasively estimated left atrial stiffness^28^. In previous papers, this echocardiographic parameter was related to LA enlargement, LV diastolic dysfunction, and collagen synthesis^27^. In a study by Kurt et al.^28^, LASI was proposed for identifying patients with diastolic heart failure. Furthermore, in physiological cardiac remodeling in athletes, as opposed to patients with cardiomyopathy, the LASI value remains low despite the large LA size^29^. Thus, it can be a parameter for differentiating physiological from pathological remodeling of the enlarged LA.

However, LAVI and LASI are co-supportive in the assessment of LA function. Yoon et al.^30^ demonstrated that LA stiffness was strongly positively correlated with LA volume indices. Noninvasively assessed LASI is also increased in patients with heart failure with preserved ejection fraction (HFpEF) and type 2 diabetes mellitus (DM) compared with those without DM^31^. Moreover, LASI was independently associated with impaired exercise tolerance (defined as an impaired VO_2_ peak and a distance walked in six minutes) and reduced quality of life (measured with the Kansas City Cardiomyopathy Questionnaire) in obese patients with HFpEF^32^. Another study showed that LASI, LAVI, and global peak atrial longitudinal strain (GPALS) independently predicted low-voltage areas in the LA assessed by electro-anatomical mapping, but LASI had the highest diagnostic accuracy for the prediction of low-voltage areas^33^. In the abovementioned study, LASI and GPALS also significantly improved the prediction of AF recurrence.

We did not find any other echocardiographic parameters to be independently predictive of impaired LAAV. However, this could be the result of some limitations of our study, and the findings of other researchers should be considered. There are reports that E′ value and E/e′ ratio, were also associated with decreased LAAV and can be useful parameters for the prediction of LAA blood stasis in patients with nonvalvular AF^24,25^. In addition, LASr, which has shown significant added value in many cardiac diseases^34^, was previously found to correlate negatively with left atrial pressures in AF patients with sinus rhythms during echocardiography^35^. It is currently being used increasingly in clinical practice, particularly in patients with AF and HFpEF. The usefulness of this parameter in the aforementioned entities is even greater, as reduced LASr values may indicate increased LV filling pressure even before the onset of left atrial enlargement^36^. Interestingly, in our present study, neither LASr nor E/e′, as stand-alone parameters, proved to be predictors of reduced LAAV, but their combination (LASI) had a strong predictive value. Interestingly, the time from AF diagnosis to ablation procedure, although longer in the group with reduced LAAV, was not significantly correlated with it. Longer time from first AF diagnosis to catheter ablation has been associated in previous studies with increased mortality from any cause in all patients with and without structural heart disease^37^. Similarly, on a large group of 2000 patients with AF undergoing pulmonary vein isolation, the association between diagnosis-to-ablation time and AF recurrence has no lower limit ("the shorter the better"), whereas little gain is to be expected beyond 36 months ("the longer the more irrelevant")^38^. It seems to us that this can be explained by the increased LA remodeling with time, as described by Leventopoulos et al.^26^, which eventually leads to the development of extensive areas of irreversible LA fibrosis, including the LAA.

Overall, the results of our study show that two noninvasive echocardiographic parameters can predict semi-invasively assessed LAAV. The first is LA enlargement, estimated as increased LAVI, which has long been a recognized factor for increased cardiovascular risk and a good parameter for estimating the effect of increased LV filling pressure on the LA. The second relatively new parameter, LASI, is related to LA reservoir function and LV filling pressure. It increases with LA remodeling and is recognized as a further significant indicator of LA function. To the best of our knowledge, this is the first study to demonstrate a relationship between LAVI and LASI assessed noninvasively with LAAV in patients with persistent non-valvular AF undergoing catheter ablation.

The present study has some limitations. First, this was a single-center study with a rather small sample size, particularly for multivariable analysis. However, the prospective nature of the study allowed for collecting planned measurements according to strict guidelines. Second, there are no clear criteria for reduced LAAV in patients with atrial fibrillation, and different cut-off points were adopted depending on the study. Third, we did not analyze follow-up data on thromboembolic events and other major adverse cardiovascular events; thus, we cannot conclude the implications of our results on patients’ long-term prognosis. An important limitation for our work is the lack of analysis of ECG parameters. Some of them, such as the analysis of the P-wave and the cycle length of AF could bring new information in combination with echocardiographic parameters. However, our assumption was to estimate the value of echocardiographic parameters, and we focused on that. Another limitation is certainly the lack of information on the antiarrhythmics taken by patients. Additionally, the patients in this study were prepared only for a first-time catheter ablation; therefore, the results may not fully represent all patients with persistent non-valvular AF. However, in our opinion, the inclusion of patients admitted for second-time ablation could bias the results, as changes in echocardiographic parameters of both LA and LAA have been reported in patients with a history of ablation^39,40^.

## Conclusion

Two noninvasive echocardiographic parameters, LASI and LAVI, were the best predictors of reduced LAAV in patients with persistent non-valvular AF undergoing TEE before catheter ablation. Other echocardiographic parameters, such as E′ value, E/e′ ratio, LA and RA areas, LAEF, LVEF, LV GLS, and LASr, were associated with reduced LAAV, although they did not have independent predictive values. Our findings suggest that, in particular, the LA stiffness index related to LA reservoir function, LV filling pressure, and LA remodeling may be most responsible for reduced LAAV. Future studies are required to prove the clinical significance of our findings.

### Supplementary Information

Supplementary Table S1.

## Data Availability

The datasets are available from the corresponding author upon reasonable request.
