# Supplementary material for: Predicting reduced left atrial appendage velocity from echocardiographic left atrial function parameters in patients with atrial fibrillation undergoing catheter ablation
Source: Sci Rep. 2024 Mar 27;14:7282. doi: 10.1038/s41598-024-57947-6 (PMC10973439; doi:10.1038/s41598-024-57947-6)
Supplement: Supplementary file 1 — Supplementary Table S1. [file 41598_2024_57947_MOESM1_ESM.docx]

Table S1. Electrophysiological data of the two study groups of patients.

| Variables | **AF group** | | |
| --- | --- | --- | --- |
|  | LAAV<25 cm/s  n=44 | LAAV≥25 cm/s  n=106 | p |
| Lov voltage areas, n (%) | 19 (67.9) _(n=28)_ | 22 (28.6) _(n=77)_ | <0.001 |
| LAPmax, median (IQR) | 31 (26-39) | 21 (17-25) | <0.001 |
| LAPmin, median (IQR) | 13.5 (10-17) | 11 (8-13) | <0.001 |
| LAPmean, median (IQR) | 22 (18-25) | 15 (13-19) | <0.001 |
| Abbreviations: AF, atrial fibrillation; LAAV, left atrial appendage emptying velocity; LAP, left atrial pressure. | | | |
